# Direct electrical quantification of glucose and asparagine from bodily fluids using nanopores

**DOI:** 10.1038/s41467-018-06534-1

**Published:** 2018-10-05

**Authors:** Nicole Stéphanie Galenkamp, Misha Soskine, Jos Hermans, Carsten Wloka, Giovanni Maglia

**Affiliations:** 1 Groningen Biomolecular Sciences and Biotechnology Institute, University of Groningen, Nijenborgh 7, Groningen, 9747 AG The Netherlands; 2 Analytical Biochemistry, Department of Pharmacy, University of Groningen, Antonius Deusinglaan 1, Groningen, 9713 AV The Netherlands

## Abstract

Crucial steps in the miniaturisation of biosensors are the conversion of a biological signal into an electrical current as well as the direct sampling of bodily fluids. Here we show that protein sensors in combination with a nanopore, acting as an electrical transducer, can accurately quantify metabolites in real time directly from nanoliter amounts of blood and other bodily fluids. Incorporation of the nanopore into portable electronic devices will allow developing sensitive, continuous, and non-invasive sensors for metabolites for point-of-care and home diagnostics.

## Abstract (web summary)

Protein nanopores are emerging as sensors for a variety of biomolecules. Here the authors develop a nanopore based on the bacterial toxin ClyA, in conjunction with binding proteins for glucose and asparagine, to detect these biomolecules simultaneously from a variety of unprocessed, diluted body fluids.

## Introduction

The ability to monitor the health of individuals that exchange information through the Internet without requiring human intervention would undoubtedly bring about a revolution in personalised healthcare. Over the past decades, the miniaturisation of electronic devices combined with recent advances in the wireless transmission of data and microfluidics have opened up new possibilities for making such devices. However, one key area that is preventing their breakthrough is the production of sensing elements for biologically relevant molecules, which can be easily interfaced with silicon-based electronics. Such elements have long lagged behind the electronic industry in terms of miniaturisation and cost reduction.

At the moment, biosensors that can be integrated in electronic devices rely mostly on the electric signal that is generated by the oxidation of a target analyte catalysed by an immobilised enzyme^1^. However, such oxidase enzymes are not ubiquitous, and today’s commercially available home diagnostic and implantable devices are entirely limited to one well-known example: the glucose sensor. However, despite the sensor has seen many improvements since it was first introduced in the early 1960s^2^, it still suffers from limitations including enzyme instability, complicated surface immobilisation procedures, baseline shifts, and does not tolerate well changes in micro-environmental factors such as changes in pH and ionic strength^3–5^.

Nanopores, which are nanometre-sized water conduits spanning biological or artificial membranes, are emerging as a new class of biosensors. When a potential is applied, the flux of ions across an individual nanopore generates an electrical signal that allows direct interfacing with electronic devices. Most notably, nanopores can be integrated into electronic devices and are now used for low-cost, high-speed, and portable sequencing of DNA^6^. Nanopores can also detect polypeptide^7,8^ biomarkers. However, their use in diagnostic devices is complicated by the low, typically picomolar, concentration of such biomarkers in biological fluids. By contrast, metabolites are highly abundant in bodily fluids, and their variation in concentration is linked to many diseases^9^. Nanopores equipped with cyclodextrins^10^, cucurbiturils^11^, or cyclic peptides^12^ may recognise metabolites. However, the detection and quantification of such analytes directly from a biological sample cannot be easily implemented because the recognition elements lack selectivity.

**Fig. 1:**
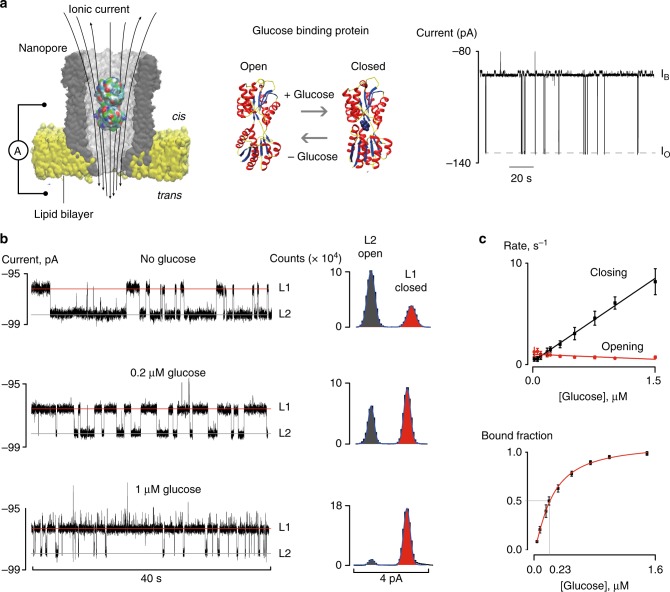
Nanopore glucose sensor for biological samples. **a** Cut-through of a surface depiction of a ClyA nanopore (grey) inserted into a lipid bilayer (yellow) containing a glucose-binding protein (GBP) created with the VMD software. GBP residues are coloured by the software according to their type. On the right are cartoon representations of GBP proteins in the open, ligand-free state and closed, liganded state. Right from the cartoon is a typical output signal (current trace) showing the entry of GBP proteins inside the nanopore. *I*_B_ is the blocked pore current signal and *I*_O_ the open pore current signal. **b** Effect of increasing concentrations of glucose added to the *trans* solution to the signal induced by GBP proteins internalised into a ClyA nanopore at −90 mV. On the left are shown current traces, on the right all-point histograms of ~50 s of a current trace. **c** Dependency of the opening and closing rates, measured as the inverse of dwell times (top) and the fraction of the closed state (bottom) on the concentration of glucose in the *trans* solution. The lines in the top graph are linear fits and the line in the graph below indicates the fitting to a Hill function. The solution used for the electrical recordings contained 150 mM NaCl and 15 mM Tris-HCl set at pH 7.5. Current traces were collected applying a Bessel low-pass filter with a 2 kHz cutoff and sampled at 10 kHz at room temperature (25 °C). A post-acquisition Gaussian filter of 200 Hz was applied. Error bars represent the standard deviation between independent experiments (*N* = 3)

Recently, we have shown that the biological nanopore cytolysin A (ClyA) can be used to monitor the function of proteins when they are lodged inside the nanopore (Fig. 1a)^13–16^. Here, using proteins from a protein family that in cells recognise an enormous variety of molecules, we show that ClyA nanopores can report the concentration of glucose and asparagine directly from samples of blood, sweat, and other bodily fluids. Conveniently, no sample preparation is required and the concentration of the metabolite can be monitored continuously.

## Results

### Characterisation of the glucose-binding protein

In this work, we used a glucose-binding protein (GBP) that binds glucose with high affinity and specificity^17^. GBP belongs to the class of substrate-binding proteins which, with minor structural differences, recognise hundreds of different metabolites in cells^18^. The structure of the GBP has been solved in the ligand-free state (open or *apo* form) configuration and in the closed, ligand-bound, configuration. Interestingly, NMR studies revealed that in solution, *apo*-GBP adopts both configurations even in the absence of glucose^19^. In our experimental setup, GBP (44 nM) was added to the *cis* side of a ClyA-AS^20^ nanopore (hereafter ClyA, Fig. 1a). A −90 mV voltage bias was then applied across the membrane, which generates an electro-osmotic flow across the nanopore promoting the entry of GBP inside ClyA, as observed by an ionic current blockade (Fig. 1a). Under these conditions, proteins remained trapped inside the nanopore for an average time of 10.1 ± 1.7 s (−90 mV, *n* = 308 events, *N* = 3 independent nanopore experiments). Two current levels (L1, *I*_res%_ = 69.1 ± 0.2%, and L2, *I*_res%_ = 67.7 ± 0.3%, with *I*_res%_ indicating the percentage ratio between the open pore current and the blocked pore current, *N* = 5, Fig. 1b) could be observed within the ionic current blockade of GBP. The relative time spent by the protein on L1 (28%) and L2 (72%) corresponded well to the percentage of closed and open configuration measured for *apo*-GBP by NMR spectroscopy (32 and 68%, respectively)^19^.

Addition of glucose to the *trans* side of the nanopore increased the number of events corresponding to the closed configuration (L1, Fig. 1b, c), while the duration of the events remained nearly constant (Fig. 1c). As expected for a binding process, the frequency of blockades scaled linearly with the concentration of the ligand (Fig. 1c). The ligand-induced closing (*k*_closing_ = 6.7 ± 0.3 × 10^6^ s^−1^ M^−1^) and opening (*k*_opening_ = 0.75 ± 0.12 s^−1^, 500 nM glucose) rate constants could be determined from the inverse of L1 and L2 dwell times (Fig. 1c), respectively, and were similar to the values measured by stopped flow (*k*_closing_ = 3–4 × 10^7^ s^−1^ M^−1^ and *k*_opening_ = 1.4 s^−1^)^21^. The dependence of the fractional time of GBP in the closed configuration with the glucose concentration fitted well to a Hill function with the Hill coefficient set to one (Supplementary Fig. 1a), from which a normalised calibration curve could be obtained (Fig. 1c). The extrapolated apparent binding constant (2.3 ± 0.1 × 10^−7^ M) corresponded well to the value measured in bulk (2–5 × 10^−7^ M)^21–23^. A GBP variant (GBP-A213R) with reduced affinity for glucose^24,25^ showed only one single level in the absence of glucose (*I*_res%_ = 67.7 ± 0.1%, *k*_closing_ = 4.69 ± 0.42 × 10^6^ s^−1^ M^−1^ and *k*_opening_ = 121.0 ± 6.9 s^−1^, Supplementary Fig. 2), corresponding to the open configuration of GBP. GBP-A213R closed at higher concentrations of glucose (20 µM, Supplementary Fig. 2). Together, these results indicate that nanopore currents can report the binding of glucose to GBP, and that the concentration of glucose inside the nanopore is similar to the concentration of glucose in bulk.

### Quantification of glucose from human biological fluids

Next, we measured the concentration of glucose directly from a panel of biological samples. Aliquots of blood (10 nL), sweat (2 µL), urine (200 nL), and saliva (8 µL) were added to the *trans* chamber of an electrophysiology setup containing a single ClyA nanopore. The untreated samples did not alter the stability of the lipid bilayer over the experimental timescale (up to 100 min, Supplementary Fig. 3). Large molecules could not enter the narrow *trans* side of the nanopore, while small molecule such as glucose could freely diffuse and bind to the trapped GBP previously added to the *cis* chamber (44 nM). Extrapolation from the calibration curve (Fig. 1c) allowed measuring the concentration of glucose directly from the diluted biological samples. Comparison with values measured with existing commercial glucose test kits showed that the nanopore accurately measured the concentration of glucose in those biological samples (Table 1 and Supplementary Table 1).

**Table 1:** Comparison of different methods measuring the concentrations of glucose and asparagine in different biological samples

	Glucose			Asparagine		
	Nanopore	Sampled volume	Commercial assay	Nanopore	Sampled volume	LC assay
Sweat	105 ± 9 µM	2 µL	105 ± 7 µM	94.6 ± 5.0 µM	5 µL	90.9 ± 4.1 µM
Urine	368 ± 7.5 µM	200 nL	381 ± 6 µM	35.4 ± 2.8 µM	4 µL	89.7 ± 3.6 µM
Saliva	5.71 ± 0.48 µM	15 µL	10.4 ± 7.9 µM	1.36 ± 0.22 µM	30 µL	ND
Blood sample 1	5.09 ± 0.88 mM	10 nL	5.3 mM	10.2 ± 0.2 µM^a^	5–15 µL	6.7 ± 0.2 µM^a^
Blood sample 2	4.91 mM	10 nL	4.9 mM			
Blood sample 3	4.21 mM	10 nL	4.4 mM			

The concentration of glucose was measured using a glucose (HK) assay kit (Sigma-Aldrich) except for blood samples, where an Accu-Chek^®^ Aviva (Roche) system was used. Since the latter system did not provide an error in the measurement, three different blood samples were tested. The concentration of asparagine was measured using a HPLC assay coupled with fluorescence detection. Sweat and urine were directly sampled, while the proteins in saliva and serum were precipitated with 8% trichloroaceticacid prior the HPLC measurements. The concentration of asparagine in saliva was too low to be measured with the HPLC assay

^a^Since a pre-purification step was required for LC sampling, serum instead of blood was used for the quantification of asparagine. Error is SD

### Detection of asparagine

**Fig. 2:**
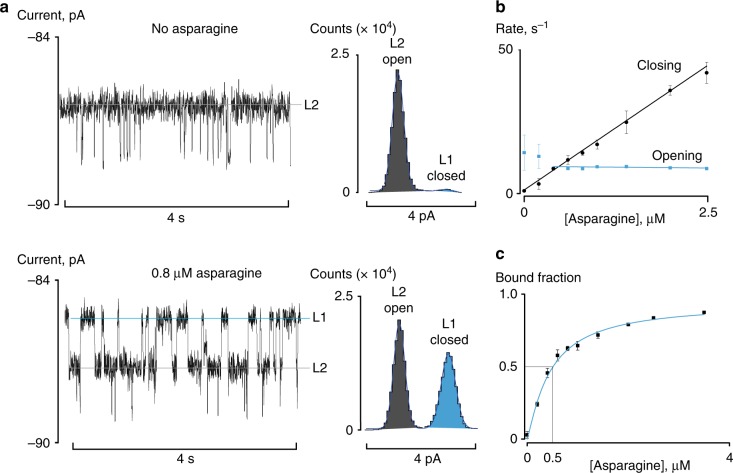
Binding constants for asparagine binding to SBD1. **a** Current traces and all-point current histograms showing the binding of asparagine to SBD1 (120 nM, *cis*) inside a ClyA nanopore. Asparagine was added to the *trans* solution and the voltage was set to −90 mV. **b** Dependency of the opening and closing rates (calculated from the inverse of the dwell times) on the concentration of asparagine in the *trans* solution. The black and blue lines are linear fits. **c** Dependency of the percentage of the closed configuration on the concentration of asparagine in the *trans* solution. The blue line indicates fitting to a Hill function with the Hill coefficient set to one. The solution used for the electrical recording contained 150 mM NaCl, 15 mM Tris-HCl, pH 7.5. Current traces were collected applying a Bessel low-pass filter with a 2 kHz cutoff and sampled at 10 kHz at room temperature (25 °C). A post-acquisition Gaussian filter of 200 Hz was applied. Error bars represent the standard deviation between independent experiments (*N* = 3)

One of the advantages of the nanopore technique is that single molecules are detected, which in turn allows the measurement of multiple analytes. To explore this concept, we used SBD1, a substrate-binding domain analogous to GBP that recognises asparagine rather than glucose. Asparagine is a marker for brain damage in acute stroke^26^, while its abnormal concentration in blood has been associated to Parkinson’s disease^27^. Under −90 mV, the blocked pore level of *apo*-SBD1 contained one main current level corresponding to the open protein configuration (Fig. 2a). The addition of asparagine to the *trans* side of the nanopore led to the appearance of an additional current level corresponding to the closed configuration of SBD1 (Fig. 2a), from which binding constants could be measured (Fig. 2b, *k*_off_ = 8.6 ± 0.1 s^−1^, *k*_on_ = 1.8 ± 0.1 × 10^7^ M^−1^ s^−1^, *K*_d_^app^ = 0.50 ± 0.04 μM). As observed before for GBP, the binding constants measured by the nanopore were similar to the ones measured in bulk^16,28^. The percentage of bound fraction was used to extrapolate the concentration of asparagine in the *trans* solution (Fig. 2c). Comparison with an established technique, high performance liquid chromatography (HPLC) coupled with fluorescence detection indicated that the nanopore approach can accurately quantify the concentration of asparagine in all biological samples (Table 1, Supplementary Fig. 4, and Supplementary Table 1). Measurements of asparagine in the presence of high concentration of glucose, glutamine, and glycine (Supplementary Fig. 5) confirmed that SBD1 shows no appreciable off-target binding activity. Measurements of asparagine in urine with known increasing amounts of asparagine (standard additions) indicated that there is no matrix effect (Supplementary Fig. 6).

### Simultaneous detection of glucose and asparagine in sweat

**Fig. 3:**
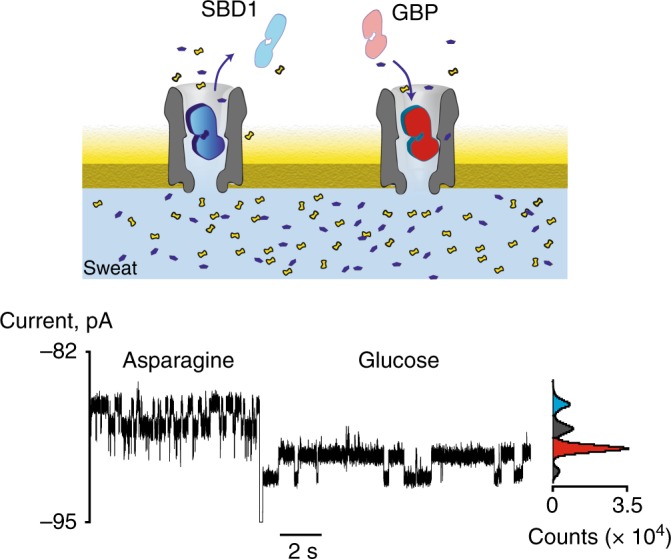
Multiplexed detection of glucose and asparagine from sweat. Schematic representation (above) and current blockades with its all-point current histogram (below) of two consecutive events showing the entry of SBD1 and GBP into a ClyA nanopore, respectively, under −90 mV applied transmembrane potential. The *trans* solution contained 5 µL of sweat constituting a 100-fold dilution. The solution used for the electrical recordings contained 150 mM NaCl and 15 mM Tris-HCl set to pH 7.5. Current traces were collected applying a Bessel low-pass filter with a 2 kHz cutoff and sampled at 10 kHz at room temperature (25 °C). A post-acquisition Gaussian filter of 200 Hz was applied

The residual current of the open configuration of SBD1 (*I*_res%_ = 67.3 ± 0.3%) was markedly different than the open protein configuration of GBP (*I*_res%_ = 69.1 ± 0.2%). Thus, when GBP (44 nM) and SBD1 (120 nM) were simultaneously present in the *cis* solution, blockades corresponding to each protein adaptor could be readily distinguished (Fig. 3), allowing the simultaneous detection of glucose and asparagine from a sweat sample (Fig. 3).

## Discussion

Portable devices for home diagnostics are sought after because they would reduce the cost of care and increase life expectancy by detecting diseases at an early stage. Here we showed that ClyA nanopores equipped with internal protein adapters can accurately measure the concentration of small metabolites by directly sampling tiny volumes of biological samples with no sample preparation. Such protein adapters are ideal biosensors, because they evolved over billion years to recognise biologically relevant molecules with high selectivity and sensitivity. Integration of ClyA nanopores with a panel of protein adapters in existing commercial portable devices would provide an off-the-shelf low-cost device for home diagnostics. The single-molecule nature of the sensor provides advantages over ensemble technologies, notably the ability of sampling of multiple analytes simultaneously without the need of calibration for signal drift. Thus, nanopores equipped with internal protein adapters are ideal next-generation biosensors for miniaturised, non-invasive diagnostic, and wearable devices.

## Methods

### Chemicals, DNA, enzymes, and lipids

Unless otherwise specified all chemicals were bought from Sigma-Aldrich. DNA was purchased from Integrated DNA Technologies (IDT), enzymes from Thermo Fisher Scientific, and lipids from Avanti Polar Lipids.

### GBP cloning

To allow cloning, a NcoI site (CCATGG) was introduced into the sequence of the wild-type GBP from *Escherichia coli* at the beginning of the gene (5′-end, beginning of the leader peptide). To maintain the open-reading frame, two extra bases were inserted after the NcoI site, resulting in an additional alanine residue after the starting methionine. For purification, a His_6_-tag was attached at the C-terminus of GBP, via a flexible glycine–serine–serine linker and the open-reading frame was terminated by two consecutive stop codons, followed by a Hind III restriction site (3′-end). The attachment of the His_6_-tag was carried out in two consecutive PCR reactions. During the first PCR reaction, the GBP gene was amplified directly from a single BL21 (DE3*) E. coli* (Lucigen) colony using Phire Hot Start II DNA polymerase, 0.4 µM dNTPs, 6 µM of MS-Glu_F (TATATATCCATGGCTaataagaaggtgataaccctgtctgctgtg), and MS-Glu_r1 (gatggtgatgGCTGCTGCCtttcttgctgaattcagccaggttgtctttatctacgcc) primers in a 50 µL reaction volume. The PCR reaction cycling protocol was as follows: pre-incubation step at 98 °C for 30 s and then 30 cycles of denaturation at 98 °C for 5 s and extension at 72 °C for 1 min. The amplified product was purified using QIAquick PCR Purification Kit (Qiagen) and served as a template for the second PCR reaction, which used ~100 ng of the purified PCR product amplified by Phire Hot Start II DNA polymerase using 0.4 µM dNTPs, 6 µM of MS-Glu_F, and MS-Glu_r2 (atatatataagctttcattaatggtGATGGTGATGGCTGCTGCCTTTCTTGCTGAATTC) primers in 300 µL volume. The cycling protocol was the same as in the previous step. The resulting PCR product encoding for the His_6_-tagged GBP gene was purified with QIAquick PCR Purification Kit (Qiagen) and digested with NcoI and HindIII (FastDigest, Fermentas). The gel purified insert (QIAquick Gel Extraction Kit, Qiagen) was cloned under control of the T7 promoter into the pT7-SC1 expression plasmid^29^ using sticky-end ligation (T4 ligase, Fermentas) via NcoI (5′) and HindIII (3′) sites. Ligation mixture of 0.6 µL was transformed into 50 µL of E. cloni^®^ 10G cells (Lucigen) by electroporation. The transformed bacteria were grown overnight at 37 °C on ampicillin-containing (100 µg/mL) LB agar plates. The identity of the clones was confirmed by sequencing.

### Protein sequence of GBP

MANKKVITLSAVMASMLFGAAAHAADTRIGVTIYKYDDNFMSVVRKAIEQDAKAAPDVQLLMNDSQNDQSKQNDQIDVLLAKGVKALAINLVDPAAAGTVIEKARGQNVPVVFFNKEPSRKALDSYDKAYYVGTDSKESGIIQGDLIAKHWAANQGWDLNKDGQIQFVLLKGEPGHPDAEARTTYVIKELNDKGIKTEQLQLDTAMWDTAQAKDKMDAWLSGPNANKIEVVIANNDAMAMGAVEALKAHNKSSIPVFGVDALPEALALVKSGALAGTVLNDANNQAKATFDLAKNLADGKGAADGTNWKIDNKVVRVPYVGVDKDNLAEFSKKGSSHHHHH

### DNA sequence of GBP

CCATGGCTAATAAGAAGGTGATAACCCTGTCTGCTGTGATGGCCAGCATGTTATTCGGTGCCGCTGCACACGCTGCTGATACTCGCATTGGTGTAACAATCTATAAGTACGACGATAACTTTATGTCTGTAGTGCGCAAGGCTATTGAGCAAGATGCGAAAGCCGCGCCAGATGTTCAGCTGCTGATGAATGATTCTCAGAATGACCAGTCCAAGCAGAACGATCAGATCGACGTATTGCTGGCGAAAGGGGTGAAGGCACTGGCAATCAACCTGGTTGACCCGGCAGCTGCGGGTACGGTGATTGAGAAAGCGCGTGGGCAAAACGTGCCGGTGGTTTTCTTCAACAAAGAACCGTCTCGTAAGGCGCTGGATAGCTACGACAAAGCCTACTACGTTGGCACTGACTCCAAAGAGTCCGGCATTATTCAAGGCGATTTGATTGCTAAACACTGGGCGGCGAATCAGGGTTGGGATCTGAACAAAGACGGTCAGATTCAGTTCGTACTGCTGAAAGGTGAACCGGGCCATCCGGATGCAGAAGCACGTACCACTTACGTGATTAAAGAATTGAACGATAAAGGCATCAAAACTGAACAGTTACAGTTAGATACCGCAATGTGGGACACCGCTCAGGCGAAAGATAAGATGGACGCCTGGCTGTCTGGCCCGAACGCCAACAAAATCGAAGTGGTTATCGCCAACAACGATGCGATGGCAATGGGCGCGGTTGAAGCGCTGAAAGCACACAACAAGTCCAGCATTCCGGTGTTTGGCGTCGATGCGCTGCCAGAAGCGCTGGCGCTGGTGAAATCCGGTGCACTGGCGGGCACCGTACTGAACGATGCTAACAACCAGGCGAAAGCGACCTTTGATCTGGCGAAAAACCTGGCCGATGGTAAAGGTGCGGCTGATGGCACCAACTGGAAAATCGACAACAAAGTGGTCCGCGTACCTTATGTTGGCGTAGATAAAGACAACCTGGCTGAATTCAGCAAGAAAGGCAGCAGCCATCACCATCACCATTAATGAAAGCTT

### Construction GBP-A213R mutant

The GBP-A213R was constructed using the QuickChange protocol for site-directed mutagenesis^30^ on a circular plasmid template DNA (pT7-SC1 containing GBP gene) with Phire^®^ Hot Start Polymerase (Finnzymes) by using two complementary primers. Six µM forward (GGTTATCGCCAACAACGATAGGATGGCA) and 6 µM reverse primer (GCGCCCATTGCCATCCTATCGTTGTT) containing a codon encoding the mutation were used in 50 µL final volume to amplify the gene (pre-incubation at 98 °C for 30 s, then 30 cycles of: denaturation at 98 °C for 5 s, extension at 72 °C for 1.5 min). The PCR product was incubated with DpnI (1 FDU) for 1 h at 37 °C to digest the plasmid template. The remaining DpnI enzyme was deactivated at 55 °C. The mixture was then incorporated into E. cloni^®^ 10G cells (Lucigen) by electroporation. Transformants containing the plasmid were grown overnight at 37 °C on LB agar plates supplemented with 100 μg/mL ampicillin and 1% glucose. Single colonies were picked and inoculated into 10 mL LB medium supplemented with 100 mg/L of ampicillin for plasmid DNA preparation. The presence of the mutation was confirmed by sequencing of the plasmid.

### Purification of SBD1

Substrate-binding domains carrying a histidine-tag were expressed in *E. coli* strain MC1061 and purified as described previously.^28^ The cell lysate was mixed with 50 mM potassium phosphate, pH 8.0, 200 mM KCl, 20% glycerol (buffer A) supplied with 20 mM imidazole and incubated with Ni^2+^-sepharose (GE Healthcare) resin for 1 h at 4 °C. Next, the resin was washed with 20 column volumes of buffer A supplied with 50 mM imidazole, followed by protein elution with 500 mM imidazole in buffer A. To prevent protein aggregation, 5 mM EDTA was added immediately after elution. Afterwards the histidine-tag was cleaved off by adding 2.5% w/w His-TEV (S219V)^31^ and dialyzing overnight against 50 mM Tris-Cl, 0.5 mM DTT, 0.5 mM EDTA, pH 8.0 to remove imidazole. The histidine-tagged tobacco etch virus (TEV) protease and residual uncut protein were removed by loading it on a small 0.5 mL Ni^2+^-sepharose column collecting the flow-through. Next, proteins were further purified with size-exclusion chromatography on a Superdex-200 column (GE Healthcare) equilibrated with 50 mM Tris-HCl, 200 mM NaCl, pH 7.5. Peak fractions were collected in aliquots and were stored at −80 °C after flash-freezing in liquid nitrogen.

### Purification of ClyA-AS

*E. cloni*^®^ EXPRESS BL21 (DE3) cells were transformed with the pT7-SC1 plasmid containing the *ClyA-AS* gene. ClyA-AS contains eight mutations relative to the *S. Typhi* ClyA-WT: C87A, L99Q, E103G, F166Y, I203V, C285S, K294R, and H307Y. Transformants were selected after overnight growth at 37 °C on LB agar plates supplemented with 100 mg/L ampicillin and 1% glucose. The resulting colonies were inoculated into 400 mL 2xYT medium containing 100 mg/L of ampicillin. The cells were grown at 37 °C (200 r.p.m. shaking) until an OD_600_ of ~0.8 was reached. The expression of ClyA-AS was induced by addition of 0.5 mM IPTG. The cell cultures were further grown overnight at 25 °C. The next day, the bacteria were harvested by centrifugation at 6000×*g* at 4 °C for 25 min and the pellets were stored at −80 °C. The pellets containing monomeric ClyA-AS were thawed and solubilized with 20 mL lysis buffer (150 mM NaCl, 15 mM Tris-HCl, pH 7.5, 1 mM MgCl_2_, 0.2 units per mL DNaseI, 10 mM imidazole, 10 µg/mL lysozyme) and incubated for 20 min at 37 °C. The bacteria were then lysed by sonication (Branson). The lysate was subsequently centrifuged at 6000×*g* at 4 °C for 20 min and the cellular debris discarded. The supernatant was mixed with 150 μL of Ni-NTA resin (Qiagen) in wash buffer (10 mM imidazole, 150 mM NaCl, 15 mM Tris-HCl, pH 7.5). After 1 h, the resin was loaded into a column (Micro Bio Spin, Bio-Rad) and washed with ~5 mL of the wash buffer. The protein was eluted with ~100 μL elution buffer (300 mM imidazole, 150 mM NaCl, 15 mM Tris-HCl, pH 7.5). Type I ClyA-AS oligomers were obtained by incubation of ClyA-AS monomers with 0.2% β-dodecylmaltoside (DDM) for 30 min at 37 °C. The oligomers were separated from monomers by blue native polyacrylamide gel electrophoresis (BN-PAGE, Bio-Rad) using 4–20% polyacrylamide gels^20^. The band corresponding to type I ClyA-AS were cut out from the gel and was extracted in 150 mM NaCl, 15 mM Tris-HCl, pH 7.5 supplemented with 0.2% DDM and 10 mM EDTA.

### Purification of GBP

*E. cloni*^®^ EXPRESS BL21 (DE3) cells were transformed with the pT7-SC1 plasmid containing the His_6_-tagged GBP gene. Transformants were selected after overnight growth at 37 °C on LB agar plates supplemented with 100 mg/L ampicillin and 1% glucose. The resulting colonies were inoculated into 400 mL 2xYT medium containing 100 mg/L of ampicillin. The cells were grown at 37 °C (200 r.p.m. shaking) until an OD_600_ of ~0.8 was reached. The expression of GBP was induced by addition of 0.5 mM IPTG. The cell cultures were further grown overnight at 25 °C. The next day, the bacteria were harvested by centrifugation at 6000×*g* at 4 °C for 25 min. The protein was purified directly afterwards by osmotic shock. The pellets were thoroughly dissolved in 20% ice-cold sucrose solution and incubated, while shaking at 4 °C for 10 min. The cell suspension was centrifuged at 6000×*g* at 4 °C for 10 min and cells collected. The cells were re-suspended in 4× volume of ice-cold MilliQ water supplemented with 5 mM MgCl_2_ and incubated, while shaking, on ice for 20 min. The cells collected again by centrifugation at 6000×*g* at 4 °C for 20 min. The supernatant was mixed with 150 μL of Ni-NTA resin (Qiagen) in wash buffer (10 mM imidazole, 150 mM NaCl, 15 mM Tris-HCl, pH 7.5). After 1 h, the resin was loaded into a column (Micro Bio Spin, Bio-Rad) and washed with ~5 mL of the wash buffer. The protein was eluted with ~500 μL elution buffer (300 mM imidazole, 150 mM NaCl, 15 mM Tris-HCl, pH 7.5). The protein (500 μL) was then dialysed exhaustively overnight against dialysis buffer (total volume of 1500 mL; 5 mM EDTA,150 mM NaCl, 15 mM Tris-HCl, pH 7.5), whereby the dialysis buffer was replaced for fresh buffer at least three times. Protein concentration was measured by using the Nanodrop 2000C at 280 nm (Thermo Fisher Scientific). Protein was stored with 10% glycerol in −80 °C after flash-freezing until further use.

### Electrical recordings in planar lipid bilayers

The setup consisted of two chambers separated by a 25 μm thick polytetrafluoroethylene film (Goodfellow Cambridge Limited) containing an aperture of approximately 100 μm in diameter. The aperture was pre-treated with a solution of hexadecane [10% (v/v) in pentane] to leave a hydrophobic coating to support bilayer formation. 1,2-diphytanoyl-*sn*-glycero-3-phosphocholine (DPhPC) in pentane (10 mg/mL) was then added to the buffer (500 µL, 150 mM NaCl, 15 mM Tris-HCl, pH 7.5) present in both compartments. After evaporation of the pentane, a lipid monolayer at the air–water interface can self-assemble spontaneously by lowering and raising the buffer across the aperture. 0.01–0.1 ng of purified ClyA-AS were added to the grounded *cis* compartment. The reconstitution of single nanopores was monitored electrically by applying a −35 mV bias. ClyA-AS nanopores display a higher open pore current at positive than at negative applied potentials, which provides a useful tool to determine the orientation of the pore. After insertion of a single pore, GBP or SBD1 were added to the *cis* compartment. This was followed by addition of a glucose or asparagine sample or bodily fluid sample in the *trans* compartment.

### Data recordings and analysis

All traces were routinely recorded at −90 mV or −60 mV applied transmembrane potential by using an Axopatch 200B patch clamp amplifier (Axon Instruments) and digitised with a DigiData 1440 A/D converter (Axon Instruments). The data was sampled by applying a 2 kHz low-pass Bessel filter and at a frequency of 10 kHz. Obtained traces were filtered digitally with a Gaussian low-pass filter with a 100 Hz cutoff. Data were recorded by using the Clampex 10.4 software (Molecular Devices) and the subsequent analysis was carried out with the Clampfit software (Molecular Devices).

### Analysis of current traces

Current transitions between the open and closed levels were analysed using the “single-channel search” function using Clampfit software (Molecular Devices). Events shorter than 2 ms were ignored. The mean dwell times for the ligand-induced closing (*τ*_on_) and ligand-independent opening (*τ*_off_) of GBP were obtained from single exponential fits to histograms of the inter-event times and dwell times, respectively. The mean lifetime of the open state (*τ*_on_) was converted to the on-rate constants by using *k*_on_ = 1/*τ*_on_[glucose], where [glucose] is the concentration of glucose added to the *trans* side. The *τ*_off_ was converted to the off-rate by using *k*_off_ = 1/*τ*_off_. The equilibrium binding constant was calculated by plotting the fraction of closed configuration against the concentration of substrate and the data fitted to a Hill function (Fig. 1c). The *K*_d_^app^ is the concentration of substrate at 50% signal saturation.

### Calibration curve for GBP binding to glucose

Values of the relative open/closed population were obtained from Gaussian fittings to all-point current histograms (Fig. 1b). In a typical experiment, an all-point histogram was created from individual current blockade longer than ~5 s. Gaussian fittings were then used to measure the area under the curve corresponding to the open and closed configuration for each protein blockade. Then, all areas were added together to calculate the overall percentage of open and closed configuration. Since the open and closed configurations were still observed in the absence of glucose or at saturated concentration of glucose (e.g., 50 µM, Supplementary Fig. 1b), the open/closed population was normalised using the following equation:

1 $$x_{{\mathrm{norm}}} = \frac{{x - x_{{\mathrm{min}}}}}{{0.94 - x_{{\mathrm{min}}}}},$$

where *x*_norm_ is the normalised value, *x*_min_ is the fraction of the *apo*-protein in the closed configuration measured during each experiment before addition of glucose, and 0.94 is the fraction of closed configuration at saturating glucose concentrations. The fraction of closed configuration was then plotted against the concentration of substrate and the data fitted to a Hill function (Fig. 1c). This graph was used as a calibration curve to determine the ligand concentration in the bodily fluids.

### Commercial glucose tests

A glucose assay kit (HK) from Sigma-Aldrich and a commercial glucose metre (Accu-chek^®^ Performa, Roche, Switzerland) were used to determine the glucose in the bodily fluids. The solution of the glucose assay kit contains a hexokinase enzyme that uses adenosine triphosphate (ATP) as a co-factor to phosphorylate glucose. The glucose-6-phosphate (G6P) is then oxidised to 6-phospho-gluconate in the presence of oxidised nicotinamide adenine dinucleotide (NAD) in a reaction catalysed by glucose-6-phosphate dehydrogenase (G6PD). The generation of NADH was spectrophotometrically observed by measuring absorbance at 340 nm. Under these conditions, the final concentration of produced NADH corresponds to the glucose concentration in the sample. In order to perform the experiment, a dilution of bodily fluid was added to the reaction mixture. The reaction was incubated for 15 min at room temperature and then the absorbance was measured at 340 nm (*ε* = 6220 M^−1^ cm^−1^). For each sample, three different dilutions were measured. The commercial glucose metre Accu-Chek^®^ uses a mutant variant of quinoprotein glucose dehydrogenase and has a measurement range between 0.6 and 33.3 mmol/L^32^. The Accu-Chek^®^ is considered to be a reliable device for monitoring glucose levels in blood from finger pricks^33^.

### Sample collection

The bodily fluids were collected from two healthy volunteers who were fasting for at least 1 h (woman in her 20’s and men in his 30’s). The blood samples were collected shortly before the experiments to minimise ex vivo artefactual changes. Sweat, urine, and saliva samples were either freshly collected before, or stored at −20 °C, before the start of experiments. The solutions were kept on ice for the duration of an experiment. The blood samples were diluted 500-folds and the urine sample 10-folds with buffer (150 mM NaCl, 15 mM Tris-HCl, pH 7.5) before adding 5 and 2 µL, respectively, to the *trans* compartment.

### Measurement time

The concentration of analytes in blood was measured by calculating the relative fraction of the open vs. closed configuration from Gaussian fittings to all-point current histograms over a certain integration time (Fig. 1b). For asparagine, the integration time was ~30 s, and for glucose ~10 min. We tested smaller integration times (Supplementary Fig. 7) and found that at least 5 min are necessary to obtain accurate measurements for glucose and about 10 s for asparagine. The difference between the two analyte lies in the different affinities of the proteins for their ligands. Glucose binds relatively strongly to GBP (*k*_opening_ = 0.75 ± 0.12 s^−1^, 500 nM glucose), compared to the binding of asparagine to SBD1 (*k*_opening_ = 8.6 ± 0.1 s^−1^, 800 nM asparagine), thus longer integration times are required for accurate reading. It follows that (GBP-A213R), which shows reduced affinity for glucose (*k*_opening_ = 121.0 ± 6.9 s^−1^ at 20 µM glucose, Supplementary Fig. 2), will provide faster reading times, with lower sensitivity, but wider detection range.

### Bilayer stability

All measurements were made by adding small volumes of biological samples to an electrophysiology chamber (500 µL). We did not observe a particular instability of the bilayer (Supplementary Figs. 3 and 4), except when lower than 1:50 or 1:30 dilutions of serum or saliva were used (Supplementary Fig. 4). Typically, with a 1:33 dilution of serum the lipid bilayer was stable for 9.4 ± 9.2 min (the error is SD, *N* = 10).

### Measurement of asparagine using high performance liquid chromatography (HPLC)

Urine and sweat samples were analysed without any pretreatment directly after the addition of 50 µL standard additions to 50 µL sample while phosphate buffered saline was used as a blank.

Proteins in 150 µL saliva and 60 µL serum samples were precipitated by incubation with 8% trichloroaceticacid (CASnr 76–03–9) for 5 min at 60 °C and 450 r.p.m. using a thermomixer (comfort, Eppendorf, Hamburg, Germany) followed by cooling on ice for 30 min and centrifugation for 10 min at 20,000×*g* and 4 °C. A fixed volume of the supernatant was taken out using a pipette not taking any precipitate and neutralised with a four times larger volume of saturated sodium borate (CASnr 1303–96–4). After mixing, a concentrator (SpeedVac 5301, Eppendorf, Hamburg, Germany) was used to evaporate all liquids by vacuum evaporation at 60 °C and 240×*g*, and the sample was reconstituted in an identical volume of water as the former fixed amount of supernatant. A final 5-min-long centrifugation at 20,000×*g* step was performed and the supernatant was stored at 4 °C before analysis.

HPLC quantification of asparagine was performed on an Agilent 1100 HPLC binary system (Agilent, Santa Clara, USA) equipped with an 1100 Fluorescence detector (FLD) and a Gemini C18 column (2 × 250 mm, 5 µm, Phenomenex, Torrance, USA) while a 40 mM sodium phosphate buffer, pH 7.8 (solvent A) and a mixture of 45:45:10 acetonitrile/methanol/water (solvent B) were used. The fluorogenic labelling of amino acids was performed automatically by the auto sampler of the HPLC machine using a mixture of 10 mg o-phtalaldehyde (OPA, CASnr 643-79-8) and 10 µL 3-mercaptapropionic acid (3MPA, CASnr 107-96-0) in water as reagent. The auto sampler mixed 0.5 µL sample with 1.3 µL 0.4 N borate buffer, pH 10.2 followed by 0.5 µL reagent and 7.6 µL water right before each injection. After the sample injection, amino acids were separated by applying solvent B at 1.5%/min increase for 25 min using a flow rate of 250 µL/min (the initial solvent B concentration was 5%). Thereafter, the column was cleaned for 5 min at 66% solvent B followed by another 12 min at 100% solvent B. Then the column was equilibrated back to 5% solvent B. Fluorescence was measured at an excitation wavelength of 340 nm and an emission wavelength of 450 nm.

Quantifications were performed using the method of standard addition at three different concentrations of analyte (10, 50, and 200 µM). The concentration of the analyte was obtained by measuring the peak areas, while each sample was analysed in triplicate. Citrulline was added as internal standard at 200 µM to correct for saliva and serum sample handling volume differences next to control run-to-run retention time shifts regarding all samples.

## Electronic supplementary material

Supplementary Information

Peer Review File

## Data Availability

The datasets generated during and/or analysed during the current study are available from the corresponding author on reasonable request.
