# Supplementary material for: Direct electrical quantification of glucose and asparagine from bodily fluids using nanopores
Source: Nat Commun. 2018 Oct 5;9:4085. doi: 10.1038/s41467-018-06534-1 (PMC6173770; doi:10.1038/s41467-018-06534-1)
Supplement: Supplementary file 1 — Supplementary Information [file 41467_2018_6534_MOESM1_ESM.pdf]

Supplementary Information

Direct electrical quantification of glucose and asparagine from bodily fluids using nanopores

Nicole Stéphanie Galenkamp[1], Misha Soskine[1], Jos Hermans[2], Carsten Wloka[1] and Giovanni Maglia[1,*]

[1] Groningen Biomolecular Sciences and Biotechnology Institute, University of Groningen, Groningen, The Netherlands

[2] Analytical Biochemistry, Department of Pharmacy, University of Groningen, Antonius Deusinglaan 1, 9713 AV Groningen, The Netherlands

* Corresponding author: Tel. +31 (0) 50 363 6138; E-mail: g.maglia@rug.nl

Supplementary Table

Supplementary Table 1.

Comparison of different methods measuring the concentrations of glucose and asparagine in different biological samples. The concentration of glucose was measured using a glucose (HK) assay kit (Sigma- Aldrich) except for blood samples, where an Accu-Chek® Aviva (Roche) system was used. Since the Accu-Chek® system did not provide an error in the measurement, three different blood sample were tested. The concentration of asparagine was measured using a HPLC assay coupled with fluorescence detection. The HPLC assay was performed directly from sweat and urine samples. Prior the HPL measurements, the proteins in saliva and serum were precipitated with 8 % trichloroaceticacid. The concentration of asparagine in saliva was too low to be measured with the LC assay.

	**Glucose**				**Asparagine**		
	**Nanopore**	**Sampled volume**	**Commercial assay**	**Sampled volume**	**Nanopore**	**Sampled volume**	**LC Assay**
**Sweat**	104 ± 14 µM	2 µl	107 ± 8 µM	50 µl	91.3 ± 2.5 µM	5 µl	130 ± 2.8 µM
**Urine**	358 ± 90 µM	300 nL	321 ± 42 µM	20 µl	32.7 ± 2.5 µM	5 µl	83.5 ± 5.2 µM
**Saliva**	13.4 ± 2.9 µM	20 µl	19.9 ± 6.8 µM	50 µl	ND	30-80 µl	ND
**Blood sample 1**	5.07 mM	40 nL	5.4 mM	~ 1 µl	4.06 ± 0.77 µM*	5-15 µl	4.2 ± 0.5 µM*
**Blood sample 2**	3.97 mM	40 nL	5.9 mM	~ 1 µl			
**Blood sample 3**	6.57 mM	40 nL	6.7 mM	~ 1 µl			

*Since a pre-purification step was required for LC sampling, serum instead of blood was used for the quantification of asparagine.

Supplementary Figures

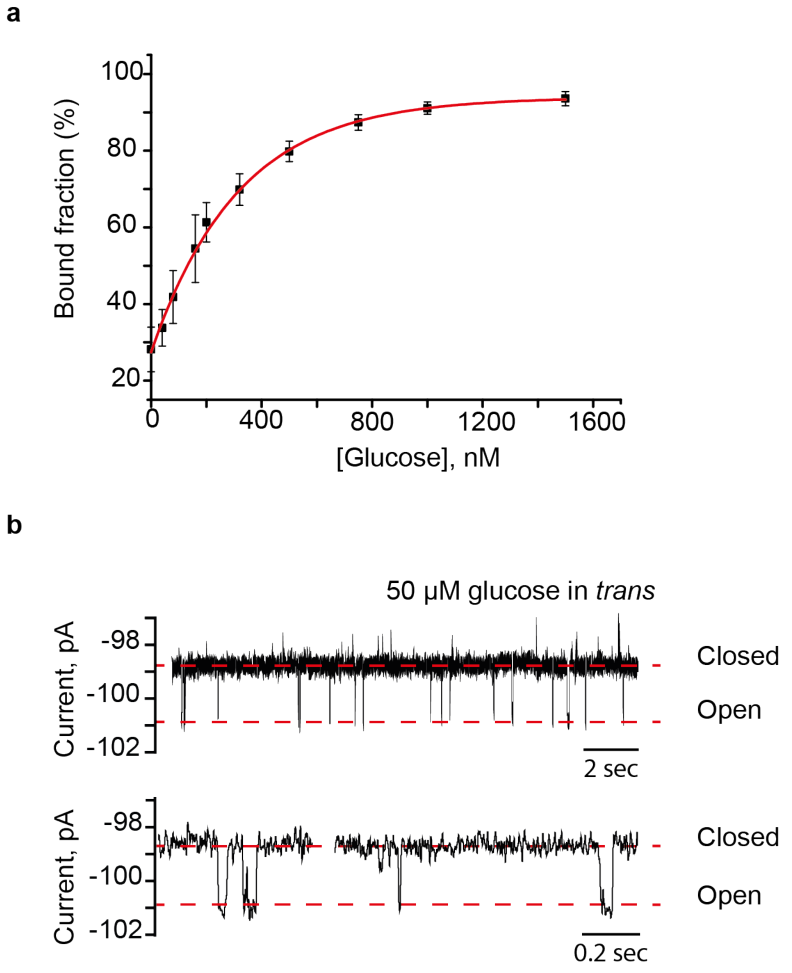

Supplementary Figure 1. Binding of glucose to GBP. **a**) Dependency of the percentage the closed configuration on the concentration of glucose in the *trans* solution. The red line indicates fitting to a Hill function with the Hill coefficient set to one. **b**) Opening and closing of GBP at a saturating concentration of glucose (50 μM, *trans*). GBP (44 nM) was added to the *cis* solution, glucose to the *trans* solution. Both solutions used for the electrical recordings were 150 mM NaCl, 15 mM Tris-HCl, pH 7.5 at room temperature (25 °C) and the applied potential was -90 mV. Current traces were collected applying a Bessel low-pass filter with a 2 kHz cut-off and sampled at 10 kHz. A post-acquisition Gaussian filter of 200 Hz was applied. Error bars represent the standard deviation between independent experiments (*N* = 3).

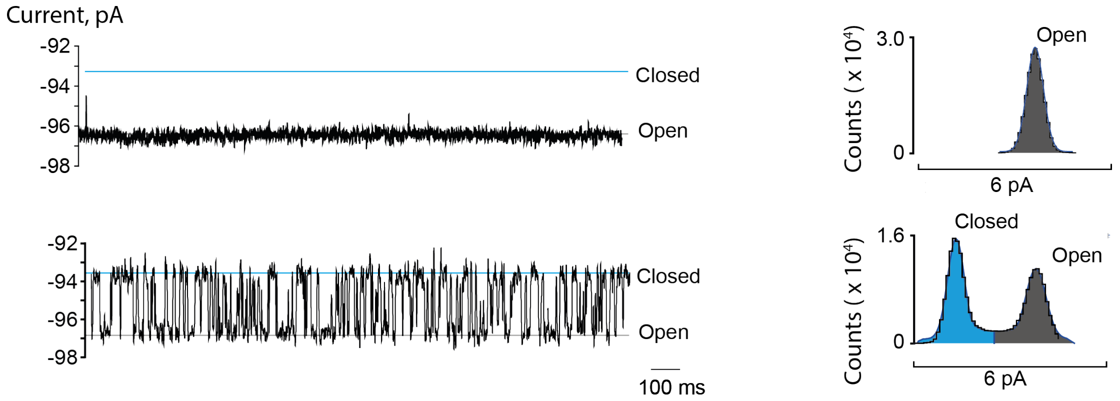

Supplementary Figure 2. Binding of glucose to GBP variant A213R. On the left a typical GBP-A213R current trace before (top) and after (bottom) the addition of 20 μM glucose to the *trans* solution. On the right are shown all-point current histograms for individual protein blockades. The voltage was -90 mV. The solution used for the electrical recordings was 150 mM NaCl, 15 mM Tris-HCl, pH 7.5 at room temperature (25 °C). Current traces were collected applying a Bessel low-pass filter with a 2 kHz cut-off and sampled at 10 kHz. A post-acquisition Gaussian filter of 200 Hz was applied.

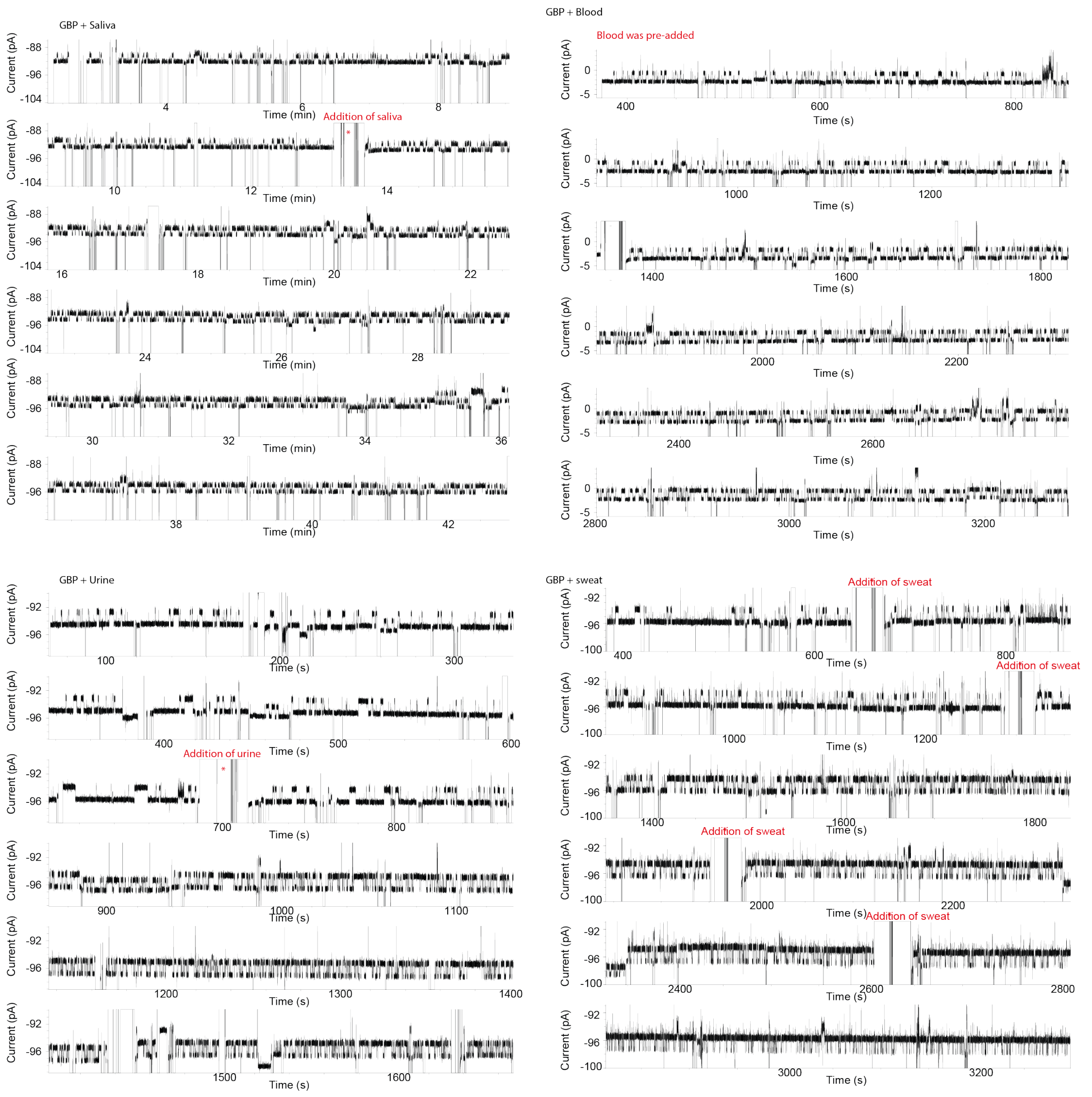

Supplementary Figure 3. **Stability of the bilayer to the addition of biological samples for glucose measurements**. The current traces show blockades induced by GBP (44 nM, *cis*) to ClyA before and / or after the addition of the biological sample to the *trans* compartment. Downward current spikes indicate the entry and exit of GBP inside ClyA. The applied potential was -90 mV. The buffered solution used contained 150 mM NaCl, 15 mM Tris-HCl, pH 7.5 and the traces were collected at room temperature (25 °C) using 2 kHz filtering and 10 kHz sampling rate. The trace was further filtered digitally with a Gaussian low-pass filter with 100 Hz cut-off. The baseline of the recording with blood was corrected using the “baseline correction” option in the Clampfit 10 software.

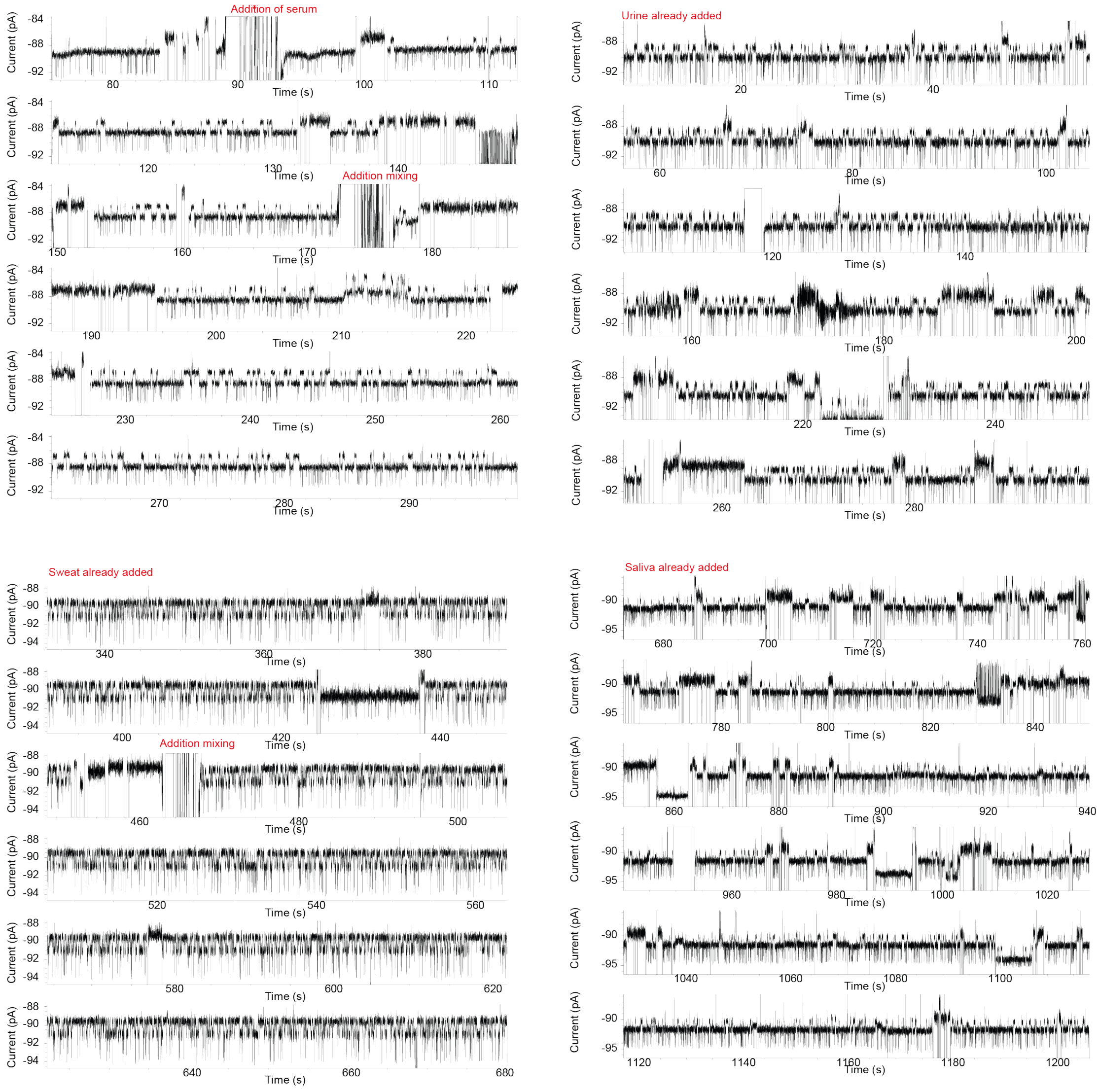

Supplementary Figure 4. **Stability of the bilayer during asparagine measurements**. The current traces show blockades induced by SBD1 (120 nM, *cis*) to ClyA. Aliquots of biological samples were added to the *trans* solution while holding the applied potential at -90 mV. The buffered solution used contained 150 mM NaCl, 15 mM Tris-HCl, pH 7.5 and the traces were collected at room temperature (25 °C) using 2 kHz filtering and 10 kHz sampling rate. The trace was further filtered digitally with a Gaussian low-pass filter with 100 Hz cut-off.

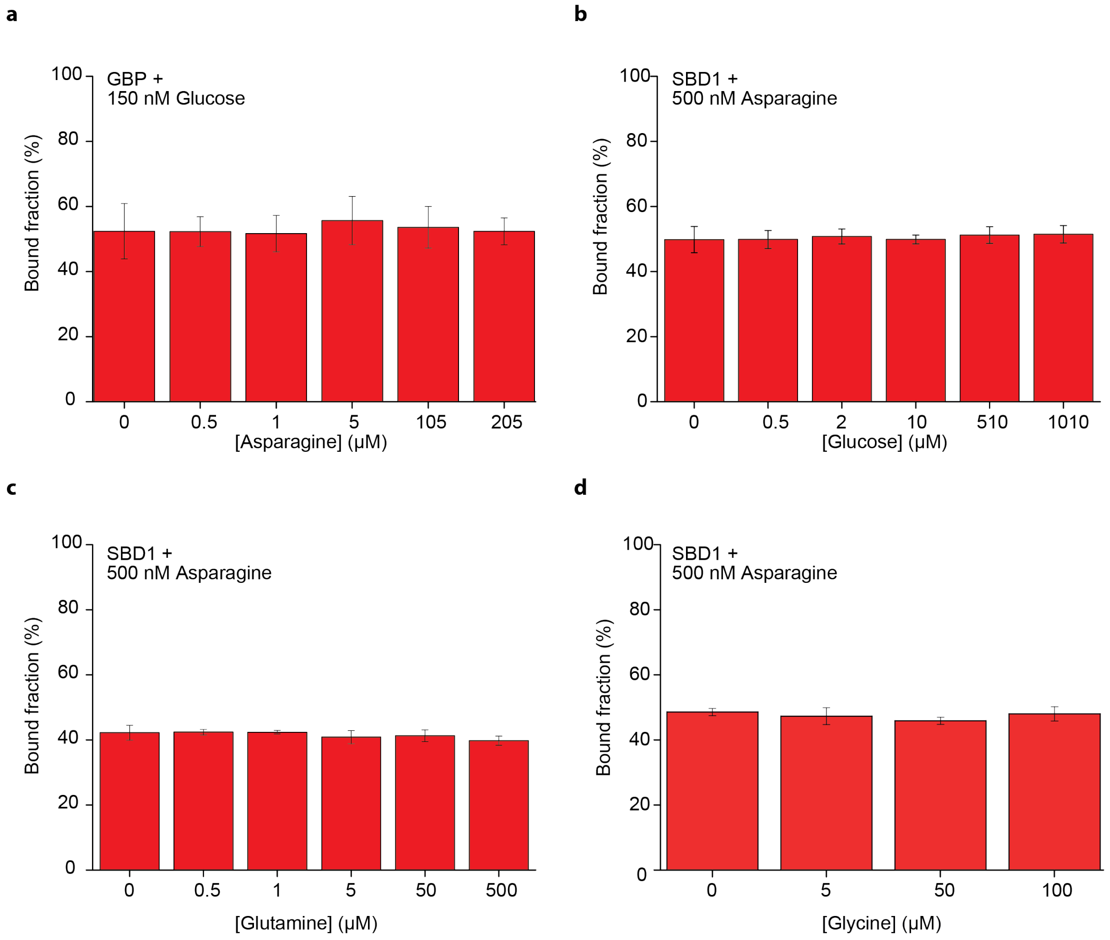

Supplementary Figure 5. Off-target binding of metabolites to SBD1 and GBP.. **a**) Histograms showing the percentage of closed state (bound fraction) of GBP in the presence of glucose and increasing concentration of asparagine. **b-d**) Histograms showing the percentage of closed state (bound fraction) of SBD1 in the presence of asparagine and increasing concentration of glucose, glutamine and glycine as indicated. The concentration of glucose (GBP) or asparagine (SBD1) was kept constant at 150 nM or 500 nM, respectively, which corresponded to the approximate K_d^{app} of GBP and SBD1, respectively. The solution used for the electrical recording contained 150 mM NaCl, 15 mM Tris-HCl, pH 7.5. All metabolites were added to the *trans* solution, while the protein was added in the *cis* solution. Current traces were collected applying a Bessel low-pass filter with a 2 kHz cut-off and sampled at 10 kHz. A post-acquisition Gaussian filter of 200 Hz was applied. Error bars represent the standard deviation between independent experiments (N = 3).

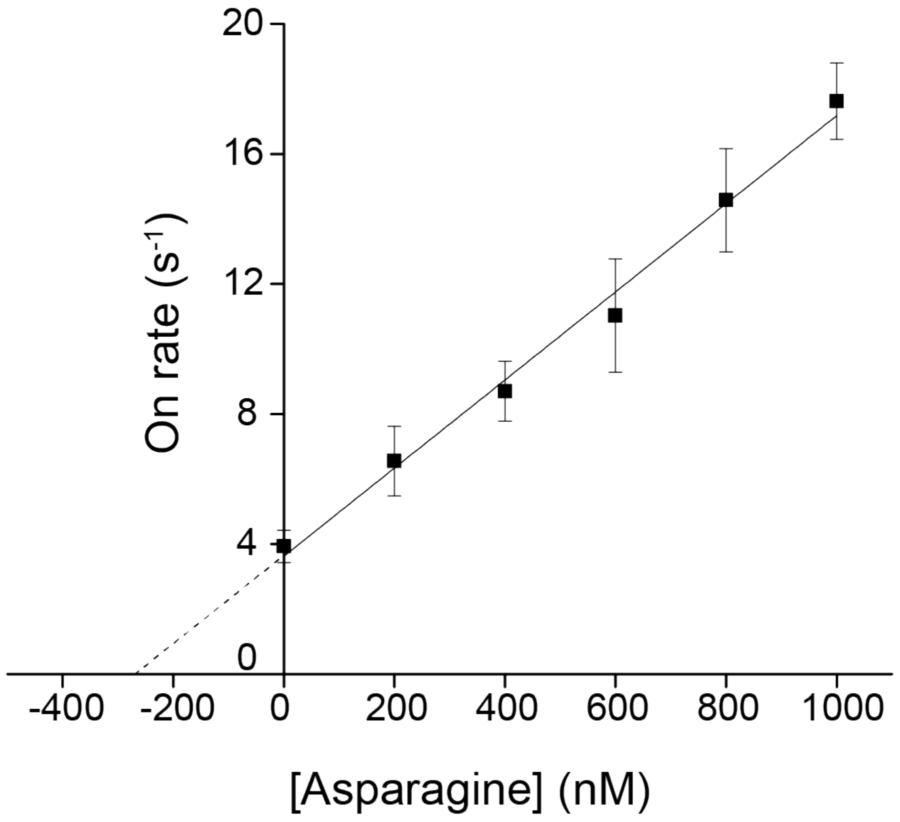

Supplementary Figure 6. Standard addition of asparagine with urine.
4 µl of urine was added to the *trans* side of a ClyA nanopore with SBD1 added to the *cis* compartment. Then aliquots of asparagine from a stock solution were added to the *trans* solution. The on rate, which was calculated from the frequency of the asparagine induced current blockades, was then measured. The extrapolated asparagine concentration in urine after standard additions (267 ± 71 nM) corresponded well to the initial value of asparagine (230 ± 12 nM), which was measured in urine from standard curves with no standard additions. Error bars represent the standard deviation between independent experiments (N = 3).

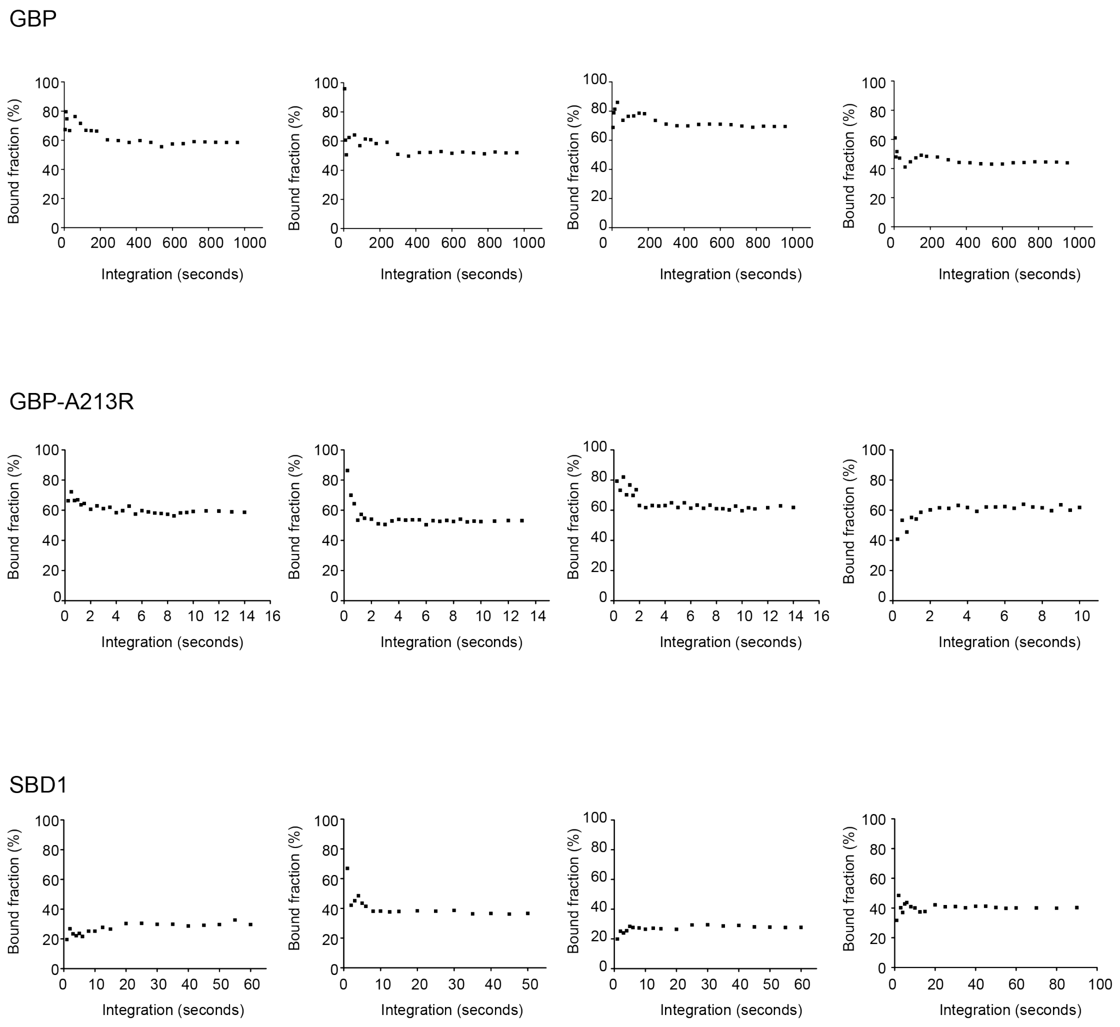

Supplementary Figure 7. **Measuring time for GBP, GBP-A213R and SBD1**. Each data point corresponds to the percentage of the closed configuration measured from full point histograms over a certain integration time for a certain fixed concentration of analyte. Fluctuations at early integration times are due to the few binding-unbinding events measured over the measurement time. GBP, which glucose release is slow, required the longest integration time, ~200 seconds. By contrast, GBP-A213R, which was engineered to release glucose very fast, required the slowest integration time, ~4 seconds.
